# GRIP: A web-based system for constructing Gold Standard datasets for protein-protein interaction prediction

**DOI:** 10.1186/1751-0473-4-2

**Published:** 2009-01-26

**Authors:** Fiona Browne, Haiying Wang, Huiru Zheng, Francisco Azuaje

**Affiliations:** 1 School of Computing and Mathematics, University of Ulster at Jordanstown, Northern Ireland, UK; 2 Laboratory of Cardiovascular Research, Research Centre for Public Health (CRP-Santé), 1A rue Thomas Edison, Strassen L-1445, Luxembourg

## Abstract

**Background:**

Information about protein interaction networks is fundamental to understanding protein function and cellular processes. Interaction patterns among proteins can suggest new drug targets and aid in the design of new therapeutic interventions. Efforts have been made to map interactions on a proteomic-wide scale using both experimental and computational techniques. Reference datasets that contain known interacting proteins (positive cases) and non-interacting proteins (negative cases) are essential to support computational prediction and validation of protein-protein interactions. Information on known interacting and non interacting proteins are usually stored within databases. Extraction of these data can be both complex and time consuming. Although, the automatic construction of reference datasets for classification is a useful resource for researchers no public resource currently exists to perform this task.

**Results:**

GRIP (Gold Reference dataset constructor from Information on Protein complexes) is a web-based system that provides researchers with the functionality to create reference datasets for protein-protein interaction prediction in *Saccharomyces cerevisiae*. Both positive and negative cases for a reference dataset can be extracted, organised and downloaded by the user. GRIP also provides an upload facility whereby users can submit proteins to determine protein complex membership. A search facility is provided where a user can search for protein complex information in *Saccharomyces cerevisiae*.

**Conclusion:**

GRIP is developed to retrieve information on protein complex, cellular localisation, and physical and genetic interactions in *Saccharomyces cerevisiae*. Manual construction of reference datasets can be a time consuming process requiring programming knowledge. GRIP simplifies and speeds up this process by allowing users to automatically construct reference datasets. GRIP is free to access at .

## Background

A fundamental problem in the post-genomic era is the determination of protein-protein interactions (PPI). Efforts have been made to map interactions on a proteomic-wide scale. Several large-scale protein-protein interaction maps have been produced for *Saccharomyces cerevisiae *(*S. cerevisiae*) and other organisms, such as *Drosophila melanogaster *and *Homo sapiens *from experimental high-throughput methods. However, even the best studied model organisms contain a large number of proteins whose interactions and functions are currently unknown [1]. This highlights the continued need for computational methods to help to direct experimentalists in the search for novel protein interactions. To validate PPI computational predictions it is essential to have a reference dataset that contains validated interactions (positive cases) and non-interacting (negative) cases. Such a knowledge reference is known as a Gold Standard and is used for prediction model construction and evaluation. Computational approaches to predicting PPIs [2,3] have utilised Gold Standard datasets to validate pair-wise protein interactions. However, there are no universal Gold Standard datasets available within the field of functional genomics and systems biology. Moreover, there is a need to share this information for supporting the implementation of more advanced computational prediction systems beyond the protein pair-wise interaction approach, e.g. module-based approaches. Myers *et al. *[4] constructed a general probabilistic system "bioPIXIE". This system provides query-based discovery of pathway-specific networks through integration of diverse genome-wide data. BioPIXIE accurately recovered known networks for 31 biological processes in *S. cerevisiae*. The Gold Standard utilized in this study was derived from the biological process Gene Ontology [5]. The Gold Standard was implemented in learning the conditional probability tables in the Bayesian network. Jansen *et al. *[2] applied a Bayesian networks approach to predicting PPI by integrating diverse genomic data. This study was extended by Lu *et al. *[3] who focused on assessing the predictive limits of genomic data integration. Gold Standards were used to validate predicted protein complexes. Positive cases were obtained from the Munich Database of Interacting Proteins (MIPS) Complex Catalogue [6]. The negative Gold Standard was obtained from pairing proteins from different compartments using *S. cerevisiae *localisation data. These studies produced accurate PPI networks providing a comprehensive view of the *S. cerevisiae *interactome. A recent study by Collins *et al. *[7] merged two affinity purification/mass spectrometry studies in *S. cerevisiae *into a single reliable collection of experimentally-based PPIs by analysing the primary affinity purification data using a novel Purification Enrichment (PE) scoring system. Using a Gold Standard obtained from MIPS [6] and *Saccharomyces *Genome Database (SGD) their study demonstrated that the consolidated dataset is of greater accuracy than the individual sets and is comparable to PPI defined using more reliable, small-scale experimental methodologies.

Information on protein interactions is usually extracted from sources such as databases to construct Gold Standards. This manual process is time consuming, complex and requires programming knowledge. We have constructed GRIP (Gold Reference dataset constructor from Information on Protein complexes) which removes the complexity of this process and reduces the time required by automatically constructing Gold Standards for researchers.

GRIP has been developed for *S. cerevisiae*, which is an important model organism for systems biology. GRIP provides three main types of functionality:

### (1) The generation of reference datasets for the inference of protein interactions

**Figure 1:**
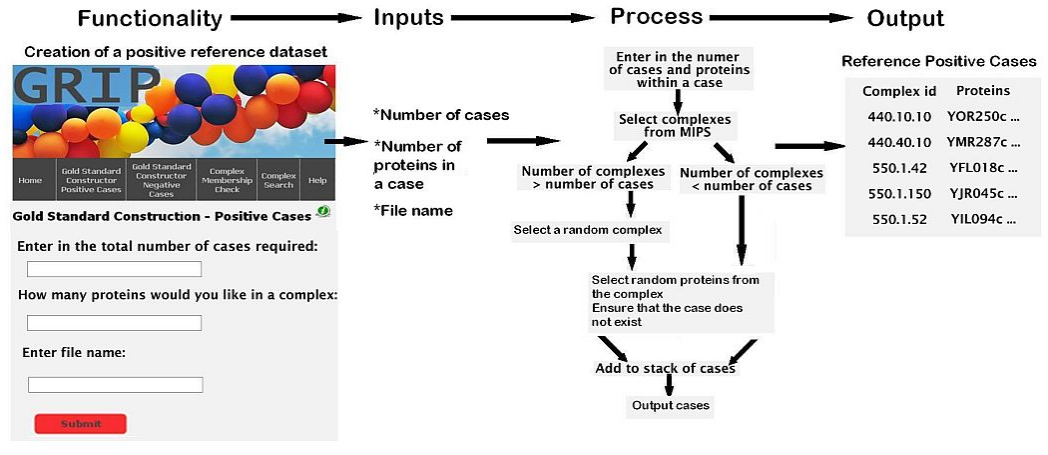
**Graphical overview of the steps taken by GRIP to produce a reference dataset**.

Both positive and negative reference datasets are constructed by GRIP. The reference datasets are constructed using data obtained from two sources: the MIPS Comprehensive Yeast Genome Database (CYGD) – Complex Catalogue and Localisation Catalogue [6] and the BioGRID repository [8]. Using the biological assumption that proteins found in the same protein complex are more likely to interact, positive cases are extracted from complex data obtained from MIPS complex catalogue. Positive cases are also attained from validated genetic and physical interactions acquired from the BioGRID repository [8]. Defining a 'negative' case is not a trivial task, some researchers may implement different criteria such as random selection of proteins [9] or generating proteins whose sub-cellular location is different [2,3]. By selecting proteins from different sub-cellular locations researchers suggest proteins are less likely to be interacting [2,3] generating high quality non-interactions. Other studies implement a simpler scheme, selecting proteins at random from a set of proteins [9]. Using protein complex and localisation data from the MIPS complex catalogue both these criteria are implemented for the construction of negative reference datasets. Using the BioGRID repository as a data source, a random selection of protein pairs are used to construction the negative reference dataset. The MIPS complex catalogue was selected as a source for Gold Standard construction as it contains lists of known protein complexes based on data collected from validated, small-scale studies obtained from the biomedical literature. Complex data labelled "Complexes by Systematic Analysis" were excluded from this study as they have not been manually verified. Locations labelled "other sub-cellular localisation", "ambiguous" and "integral membrane/endomembranes" were excluded as these data are generated using high-throughput analysis or these location labels are not adequately specific for inclusion in GRIP. In addition the locations "extracellular", "cell wall", "cell periphery" and "plasma membrane" are grouped together into one location. The BioGRID repository was selected as a source as the data is obtained from validated genetic and physical interactions extracted from literature [8]. A user can select either the MIPS or BioGRID as the data source to construct a reference dataset. The reference dataset offered to the user consists of a number of cases. Using the MIPS as the data source, each 'positive' case contains the protein complex name and a list of proteins belonging to that complex (Figure 1). GRIP defines a positive case by only considering proteins in the same subclass (at the lowest level). A 'positive' case when selecting the BioGRID as the data source will contain a pair of proteins which have been validated as having a physical or genetic interaction. Different criteria have been suggested for defining a 'negative' case [2,3,9]. GRIP offers two generation criteria for constructing 'negative' cases for the MIPS data source. Firstly a negative case can be defined as consisting of a list of proteins obtained from different sub-cellular locations and complexes. Secondly a negative case can be defined as consisting of a list of proteins randomly selected from sub-cellular locations and complexes. GRIP provides users with the flexibility to determine the number of proteins in a given case. If a user stipulates that a case should consist of, for instance, two proteins, GRIP will retrieve two proteins that are found within the same complex (complexes considered have a minimum size of 5 proteins). For the BioGRID data source a 'negative' case is defined by the random selection of protein pairs. The user has the freedom to determine the total number of cases in the reference dataset.

### (2) Protein complex membership verification

GRIP provides the functionality to verify protein complex membership. Using a Web browser, a user can upload a text file containing a defined set of proteins to GRIP. Validation is performed by the system to ensure that a text file containing proteins has been submitted by the user. These proteins are validated against known protein complexes from MIPS stored in MySQL database tables. SQL queries are implemented to perform the search function. All protein complexes that contain one, some or all of the proteins in the user defined text file are selected. Using this information, complex ids and proteins found within each complex id are returned. These results are displayed on the GUI and a download facility is provided to the user whereby results can be downloaded as a text file. If no matches are obtained by the search function, a message is displayed to users on the GUI.

The protein complex membership verification is a valuable function for users to validate possible protein complex membership predictions. GRIP not only returns the complex details in which all the proteins may exist, it also returns complexes where one or some of the defined proteins exist.

### (3) Protein complex matching

GRIP provides the utility to search and retrieve protein complex information related to a complex id or complex name (as defined by the MIPS complex catalogue) input by the user. GRIP accepts either protein complex name or complex id as a search term. The complex matching algorithm will search for protein complexes containing the search term. The protein complex search function provides the flexibility of allowing partial names and ids to be entered. Using SQL queries, protein complex information stored in MySQL database tables are scanned to match the search term. If matches to the search term are found, complex description, complex id and protein information are displayed via the GUI. A message on screen also informs the user if no results are obtained.

The functionality for constructing a reference dataset, protein complex membership and protein complex matching has been integrated into a web-based system. To the best of the authors knowledge, no tool currently exists that incorporates together these different functions. Additionally the user has the option of construct a reference dataset from either the MIPS complex catalogue or BioGRID repository. GRIP is a useful resource for the task of computational inference of PPI networks in *S. cerevisiae*. Classification techniques have been implemented to integrate diverse sources of functional genomic data for the computational prediction of PPI networks [2,3]. Reference datasets are required to either train or to assess the predictive quality of the classification methods implemented. Predicted PPI can be verified in GRIP using the protein complex verification tool.

## Implementation

GRIP provides a GUI interface which can be used to construct reference datasets for computational PPI prediction, verifying predictions and searching for protein complex information. The GRIP tool has been developed using the PHP programming language [10] in an object-oriented approach to allow reuse of coded modules. The MIPS CYGD Complex Catalogue and Localisation Catalogue are stored in a MySQL relational database management system [11]. GRIP runs on an Apache Web server platform [12]. It is possible for GRIP to be implemented on different CGI servers and databases. Development was performed using MySQL and Apache as both these resources are open source and freely available. Files are uploaded and downloaded as plain text files. No installation is required by the user, the GRIP system can be accessed via a Web browser.

## Results and discussion

### Construction of reference datasets

The reference dataset constructor provides the functionality to create positive and negative cases. For creating either a positive or negative reference dataset, users can select the number of cases required (between 1 and 10000). Initially an upper limit of 10000 has been placed on number of cases due to an increase in processing time. Users can download the complete positive and negative reference dataset for pair-wise interactions from either the MIPS complex catalogue or BioGRID. The number of proteins (between 2 and 11) to be included (i.e. pair-wise interactions or module-based interacting proteins) can also be selected when the MIPS complex catalogue is selected as the data source. Once GRIP has constructed the reference dataset, the results can be viewed in the browser or downloaded to users' local machine.

#### Positive Cases

Using the MIPS complex catalogue as a data source, GRIP constructs a positive dataset by performing a search to find complexes that contain a number of proteins greater than or equal to *n *(where *n *is the number of proteins required for a case). A positive case consists of proteins which belong to the same complex. Only MIPS complex sub-classes are considered. For instance, the MIPS class 'Transcription complexes/Transcriptosome' contains the subclasses 'mRNA guanylyl transferase', the 'DNA repair complexes' and other subclasses related to transcription complexes. Complexes that match these criteria are randomly sampled to ensure that no bias occurs in selecting the complexes. Proteins from each complex will be randomly chosen to form a case. Users can select the BioGRID as the data source for constructing a positive reference dataset. Only protein pairs that have been verified as having a genetic or physical interaction are considered as 'positive' cases. To use the Gold Standard Constructor for Positive Cases, a user firstly selects the data source (MIPS complex catalogue or BioGRID). Secondly the user enters the number of positive cases they require in a positive dataset. The number of cases can lie between 1 and 10000. If the user selects the MIPS complex catalogue then the number of proteins that make up a case can be entered. This number must lie between 2 and 11. The positive cases are written to a text file which can be viewed in the browser or downloaded to the user's computer. Additional checks are performed to ensure that two cases will not contain the same proteins. Therefore, the user obtains a dataset containing unique cases. Figure 1 provides a graphical overview of the generation of a positive dataset using the MIPS complex catalogue as the data source.

#### Negative Cases

For the construction of a negative reference datasets the user has the option to select either the MIPS complex catalogue or BioGRID as the data source. To construct a negative case using the MIPS complex catalogue as the source, a user can select from two different generation criteria. For the first criteria – different sub-cellular locations and complexes, GRIP randomly selects proteins belonging to eleven different sub-cellular locations (mitochondria, nucleus, cytoplasm, endosome, endoplasmic reticulum, transport vesicles, cytoskeleton, cell wall, golgi, vacuole, bud). Further analysis is performed by GRIP to ensure that each protein in a case belongs to a different complex and each case is unique. Each protein is required to come from different sub-cellular locations and complexes when constructing negative cases for the pair-wise and module-based approaches. The second criteria – randomisation, GRIP randomly selects proteins in the eleven sub-cellular locations. When the BioGRID is selected as the source, random protein pairs are selected by GRIP. To use the Gold Standard Constructor to create negative cases, a user firstly enters the total number of negative cases that they require in the dataset. The number of cases must lie between 1 and 10000 (system initial setting). The user then enters the number of proteins in a case if the MIPS complex catalogue is the data source. This must lie between 2 and 11. The negative cases are written to a file which can be viewed in the browser or downloaded to the user's computer. Additional checks are performed to ensure each case is unique and each case contains unique proteins.

### Protein Complex Membership Matching

**Figure 2:**
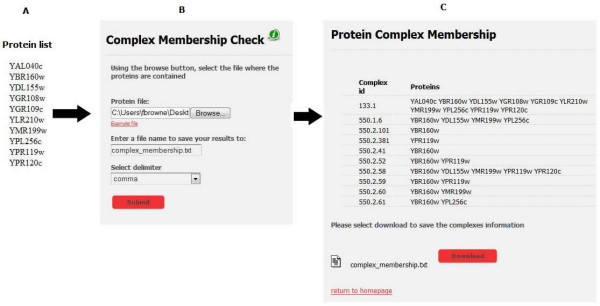
**Graphical overview of the steps involved when verifying protein complex membership**. A. The user constructs a list of proteins they wish to check for protein complex membership. B. The protein list is uploaded to the server and the name of the results file is specified. C. Using coded protein complex membership function GRIP determines protein complex membership and search if the proteins exist in the same complex. The results obtained are displayed on a GUI and a text file of results can be downloaded.

Figure 2 illustrates the GRIP functionality to verify protein complex membership (data obtained from the MIPS complex catalogue). The protein complex membership matching function is encoded in PHP and includes the use of SQL statements to query the database tables containing protein complex information. The protein list from the user is uploaded onto the GRIP Web-server. From here PHP code places the proteins from the user's text file into an array. For each protein in the array, an SQL query is performed to find the complexes in which these proteins exist. Once all unique protein complexes are obtained, further SQL queries are employed to extract the proteins from the complexes. Both the complex id and proteins are returned by the system.

### Protein Complex Matching

**Figure 3:**
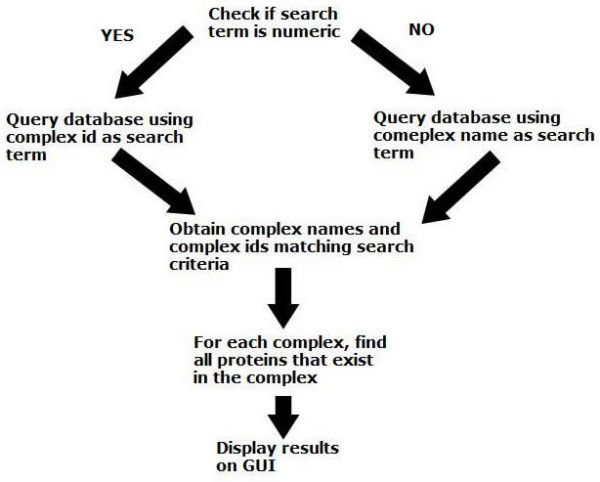
**Description of the complex matching algorithm used to search for complexes and proteins matching a client's search term**.

Figure 3 illustrates the complex matching algorithm whereby GRIP searches for protein complexes containing the search term entered using the GUI. The PHP algorithm firstly determines if the search criteria entered by the user is a complex id or complex name. The search criteria are inserted into an SQL query to obtain complex information related to the search term. For each of the protein complexes obtained, proteins within the complex are also extracted. All these data are output to the GUI.

### Evaluation of the Reference Dataset

**Figure 4:**
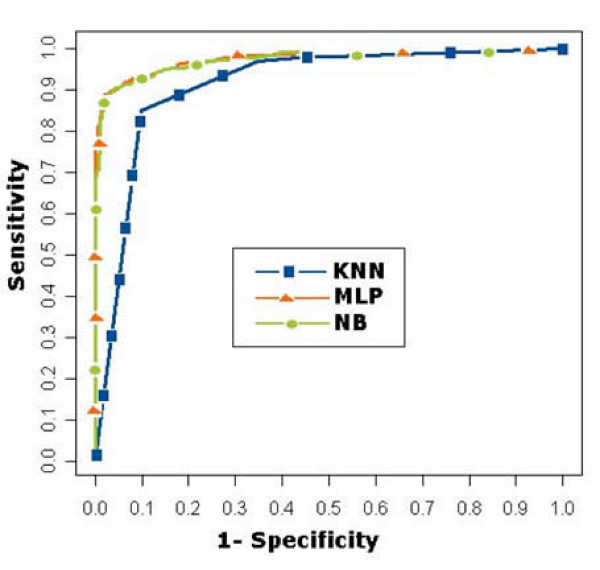
**ROC curves produced by the classifiers NB, MLP and KNN when integrating seven features for (a) pair-wise prediction of PPI network (b) module-based prediction of PPI network**.

Previous work by [13] evaluated three classification techniques Naive Bayesian (NB), Multi-Layer Perceptron (MLP) and K-Nearest Neighbour (KNN) for the predictive task of inferring pair-wise and module-based PPI interaction networks in *S. cerevisiae*. Seven functional genomic data ranging from co-expression to essentiality were integrated using the classification techniques. The predicted interactions were verified using a reference dataset constructed by GRIP as the MIPS complex catalogue as the data source. The reference dataset consisted of 10802 positive cases (which is consistent with positive cases generated in a previous study by Collins *et al. *[7]) and 330,000 non-interacting cases. These numbers were selected as they are the maximum number of pair-wise interactions that could be obtained from the MIPS complex catalogue. Classification performance was evaluated using Receiver Operating Characteristic (ROC) curves. ROC curves plot the sensitivity against (1 - specificity) obtained by the classifiers as the discrimination threshold is varied. A perfect classifier will obtain an ROC curve that rises steeply along the left axis to the point (false positive rate equals 0, true positive rate equals 1). An uninformative classifier will produce a ROC curve that is a diagonal 45 degree line. AUC is the measurement of the total area under the ROC curve. Figure 4 illustrates ROC curves obtained by each individual classifier when integrating all seven genomic features for the prediction of pair-wise and module-based interactions in *S. Cerevisiae *using 10-fold cross validation. From Figure 4 it is observed that the NB and MLP obtain a marginally higher AUC values compared to the KNN classifier. Additional information on the prediction of pair-wise and module-based PPI networks can be found in the study [13].

## Conclusion

GRIP integrates the functionality for constructing reference datasets, protein complex membership matching and protein complex matching into one public, user-friendly environment. Currently, no other tool exists that performs all these functions. GRIP provides the user with two validated data sources (either MIPS complex catalogue or BioGRID) to construct the reference datasets. GRIP has been developed as a web-based system to allow ease of access and sharing of these resources. GRIP can support the development and evaluation of computational models for PPI prediction as it provides researchers with a resource to create reference datasets (Gold Standards). The automatic construction of these reference datasets removes programming required thereby simplifying and saving time for researchers. Recent research by [2,3,13] demonstrated that the generation of reference datasets are critical for the verification of computationally-inferred PPI networks. A study by [13] implemented reference datasets constructed using GRIP to demonstrate that supervised statistical and machine learning techniques can be successfully applied to pair-wise and module-based interaction prediction.

We plan to extend GRIP to provide Gold Standard datasets for other organisms, including humans. Future versions of GRIP will allow users to construct Gold Standards using additional diverse, validated reference datasets.

## Server version requirements

**Project Name**: GRIP

**Project Home Page**: 

**Operating system(s)**: Platform independent

**Programming language**: PHP

**Other Requirements**: Apache server, Linux Server, MySQL sever

**Restrictions**: None

## Client version requirements

**Requirements**: Web-browser (such as Internet Explorer, Mozilla Firefox), internet connection.

## Abbreviations

GRIP: Gold Reference dataset constructor from Information on Protein complexes; GUI: Graphical User Interface; KNN: K-Nearest Neighbour; MLP: Multi-Layer Perceptron; NB: Naïve Bayesian; PHP: Hypertext Pre-Processor; PPI: Protein-Protein Interactions; SQL: Structured Query Language.

## Competing interests

The authors declare that they have no competing interests.

## Authors' contributions

FB developed the source code of the GRIP Web-system. HW, HZ and FA contributed to the system specification, design and evaluation. All authors contributed towards the manuscript and documentation. All authors read and approved the final manuscript.
